# "Acute pseudo-pericardial tamponade": the compression of the thoracal inferior vena cava – a case report

**DOI:** 10.1186/1749-8090-4-9

**Published:** 2009-02-24

**Authors:** Raif Cavolli, Kaan Kaya, Altay Omer Elalmis

**Affiliations:** 1 GOP University, Medical School, Cardiovascular Surgery Department, Tokat, Turkey

## Abstract

We describe a case of 68-year-old woman which was admitted to our hospital for mitral valve replacement (MVR), in whom acute compresion of the vena cava inferior developed after repair of lacerated atrio-caval junction with hemostatic tissue sealant, biologic glue (BioGlue, Cryolife, ınc, Kennesaw, Ga). Removal of the BioGlue relieved the unexpected problem.

## Background

Compression of intrapericardial structures is well-known issue in cardiac surgery. Restriction of the heart by a localized mass in the pericardial space may cause atypical findings of compression.

Cardiac tamponade can be caused not only by ventricular compression [1]. Isolated compression of the inferior vena cava by haemostatic tissue sealant, biologic glue (BioGlue) has never been reported yet.

Here, we report a case who suffered from acute heart failure early after operation (mitral valve replacement) due to compression of the thoracal vena cava inferior by haemostatic tissue sealant, biologic glue (BioGlue), in which clinic features of the patient mimics the pericardial tamponade.

## Case presentation

A 68-year-old woman was admitted to our hospital for mitral valve replacement (MVR). The mitral valve was approached from the right side through an incision in the left atrium posterior to Waterson's groove. Related to small left atrium and insufficient exposure of mitral valve due to excessive traction of the left atrium, resulted in laceration of the vena cava inferior-right atrial connection (IVC). After mitral valve replacement (27 mm Sorin) we closed the left atrium and repaired the injured IVC. Multiple attempts at primary suture repair were made but due to extreme friability and brittleness, and to minimize the bleeding we decided to use the haemostatic tissue sealant, biologic glue (BioGlue). The bleeding stopped. And the patient was transferred to the cardiac intensive care unit without any problem. During the following hour patient's blood pressure were labile without a change of the central venous pressure (CVP) measured from the right internal jugular vein. Postoperative second hour patient's blood pressure decreased to 70/40 mmHg and heart rate increased 135 per minute. At this period urine output decreased too. At this time we didn't record any increasing at CVP values (CVP was 6 mmHG). Chest X-Ray didn't showed enlargement of cardiac silhouette. The repeated echocardiography revealed a minimal amount of the pericardial effusion on the four chamber view (without any compression of the right ventricle or right atrium was not evident at this moment), good cardiac function, and relatively under-filled volume status. The ECG displayed no signs of ischemia. As haemodynamic stabilisation was not achieved by volume supply and inotropic support, urgent surgical management was intended. The chest was opened emergently, even though right atrial pressure tracing did not suggest cardiac tamponade.

**Figure 1:**
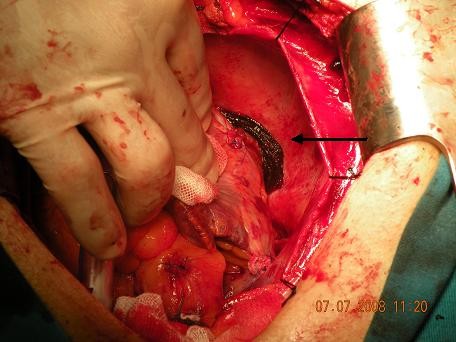
**Intraoperative view of compression of the vena cava inferior by the bioglue with absorbable haemostat (surgicell)**.

No significant pericardial effusion (or blood clot) was found. After detailed exploration we decided to remove the bioglue from the around the VCI (Fig 1). After removing the bioglue we achieved a prompt disappearance of the symptoms (heart rate was decreased from 135 to 85 beats/min and blood pressure increased to 110/70 mmHg, and all inotropes were stopped). The sternum was closed and the patient was transferred to the cardiac intensive care unit. The postoperative course was uneventful and the patient was discharged on the postoperative day 7.

## Conclusion

Cardiac tamponade is a potentially life-threating condition that requires prompt recognition and intervention [2]. The ability to establish the correct diagnosis necessitates integration of symptomatic manifestation, physical signs, and imaging features [3]. Conventional trans-thoracic echocardiography does not always provide good image quality soon after open-heart surgery because the transducer is placed near the surgical wound. Furthermore, mechanical ventilation or the air trapped in the thoracic cavity interferes with ultrasound penetration. It is especially difficult to observe the right side of the heart. When pericardial mass is loculated in an unusual location, it can be more challenging to make diagnosis when loculated effusion (or any mass in pericardial space) is inaccessible to the TTE ultrasound beam [4]. For technical reason the use of the transesophageal echocardiogram was impossible in this case. This is a limitation of our case report.

A loculated, eccentric effusion or localized hematoma can produce regional tamponade in which only selected chambers are compressed. As a result, diagnosis is challenging because the typical physical, hemodynamic, and echocardiographic signs of tamponade are usually absent.

BioGlue is a preparation of bovine albumin cross-linked with glutaraldehyde to form a strong adhesive bond. It is supplied in a 2-barrel applicator containing albumin in 1 barrel and glutaraldehyde in 1 barrel, which are mixed at the time of application. After mixture, the glue solidifies in approximately 2 to 3 minutes [5]. Some experimental and clinical studies have reported the effect of BioGlue in creating local tissue damage and local tissue damage and local inflammatory response, and increasing risk of pseudoaneurysm formation and great vessel stenosis especially in pediatric population [6]. There was a case report of superior vena cava stenosis as the result of an inappropriately large amount of BioGlue placed between the roof of the left atrium, the superior vena cava, and the aortic root for persistent bleeding [7]. This case was an example of regional cardiac tamponade. To our knowledge this is the first reported case of an external compression of the vena cava inferior (by the BioGlue) imitating similar symptoms and the haemodynamic consequences of a pericardial tamponade. In regard to patient management, we believe that prompt exploration was an appropriate step in addressing of patient's critical condition.

## Competing interests

The authors declare that they have no competiting interests.

## Authors' contributions

RC conceived the study. KK prepared the figures. RC and AOE did the background literature search. RC was the cardiac surgeon. All authors have read and approved the final manuscript.

## Consent

Written informed consent was obtained from the patient for publication of this case report and the accompanying image. A copy of the written consent is available for review by the Editor-in-Chief of this journal.

## References

[B1] Saito Y, Donohue A, Attai S, Vahdat A, Brar R, Handapangoda I, Chandraratna AP (2008). The syndrome of cardiac tamponade with small pericardial effusion. Echocardiography.

[B2] Kuvin JT, Harati NA, Pandian GN, Bojar MR, Khabbaz KR (2002). Postoperative cardiac tamponade in the modern surgical era. Ann Thorac Surg.

[B3] Gold MM, Spindola-Franco H, Jain VR, Spevack DM, Haramati LB (2008). Coronary sinus compression: An early computed tomographic sign of cardiac tamponade. J Comput Assist Tomogr.

[B4] Beppu S, Tanaka N, Nakatani S, Ikegami K, Kumon K, Miyatake K (1993). Pericardial clot after open heart surgery: its specific localization and haemodynamics. European Heart Journal.

[B5] Karimi M, Kerber RE, Everett JE (2005). Mechanical aortic valve malfunction: An intraoperative BioGlue complication. J Thorac Cardiovasc Surg.

[B6] Kazui T, Washiyama N, Bashar AH, Terada H, Suzuki K, Yamashita K, Takinami M (2001). Role of biologic glue repair of proximal aortic dissection in development of early and midterm redissection of aortic root. Ann Thorac Surg.

[B7] Economopoulos GC, Dimitrakis GK, Brountzos E, Kelekis DA (2004). Superior vena cava stenosis: a delayed BioGlue complication. J Thorac Cardiocasc Surg.

